# Histographic analysis of oedema and fat in inflamed bone marrow based on quantitative MRI

**DOI:** 10.1007/s00330-020-06785-x

**Published:** 2020-04-14

**Authors:** Timothy J. P. Bray, Naomi Sakai, Alexandra Dudek, Corinne Fisher, Kannan Rajesparan, Andre Lopes, Coziana Ciurtin, Debajit Sen, Alan Bainbridge, Margaret A. Hall-Craggs

**Affiliations:** 1 Centre for Medical Imaging, University College London, London, UK; 2 Arthritis Research UK Centre for Adolescent Rheumatology, University College London, London, UK; 3 Cancer Research UK & University College London Cancer Trials Centre, London, UK; 4 Medical Physics Department, University College London Hospitals, London, UK

**Keywords:** Magnetic resonance imaging, Inflammation, Arthritis, Spine

## Abstract

**Objective:**

To demonstrate proof-of-concept for a quantitative MRI method using histographic analysis to assess bone marrow oedema and fat metaplasia in the sacroiliac joints.

**Materials and methods:**

Fifty-three adolescents aged 12–23 with known or suspected sacroiliitis were prospectively recruited and underwent quantitative MRI (qMRI) scans, consisting of chemical shift-encoded (at 3 T) and diffusion-weighted imaging (at 1.5 T), plus conventional MRI (at 1.5 T) and clinical assessment. qMRI scans produced proton-density fat fraction (PDFF) and apparent diffusion coefficient (ADC) maps of the sacroiliac joints (SIJs), which were analysed using an in-house software tool enabling partially automated ROI definition and histographic analysis. Logistic regression and receiver operating characteristic (ROC) analyses assessed the predictive performance of ADC- and PDFF-based parameters in identifying active inflammation (oedema) and structural damage (fat metaplasia).

**Results:**

ADC-based parameters were associated with increased odds of oedema (all *p* < 0.05); ROC-AUC was higher for histographic parameters representing the upper end of the ADC distribution than for simple averages. Similarly, PDFF-based parameters were associated with increased odds of fat metaplasia (all *p* < 0.05); ROC area-under-the-curve was higher for histographic parameters representing the upper end of the PDFF distribution than for simple averages. Both ADC- and PDFF-based histographic parameters demonstrated excellent inter- and intra-observer agreement (ICC > 0.9).

**Conclusions:**

ADC-based parameters can differentiate patients with bone marrow oedema from those without, whilst PDFF-based parameters can differentiate patients with fat metaplasia from those without. Histographic analysis might improve performance compared with simple averages such as the mean and median and offers excellent agreement within and between observers.

**Key Points:**

*• Quantitative MRI with histographic analysis can identify bone marrow oedema (an active inflammatory lesion) and fat metaplasia (a ‘chronic’ inflammatory lesion) in patients with spondyloarthritis.*

*• The use of histographic analysis might improve the performance of quantitative MRI for detecting bone marrow oedema and fat metaplasia compared with simple averages such as the mean and median.*

*• Bone marrow oedema and fat metaplasia are known to be of diagnostic and prognostic significance, and the proposed method could support clinical decisions around biologic (and other) therapies in spondyloarthritis.*

**Electronic supplementary material:**

The online version of this article (10.1007/s00330-020-06785-x) contains supplementary material, which is available to authorized users.

## Introduction

Spondyloarthritis (SpA) encompasses a group of immune-mediated inflammatory diseases characterised by spinal pain, stiffness and damage which commonly affect young people and have poor long-term health outcomes [1]. Diagnosis of SpA is often difficult due to the complex nature of pain in young patients [2], and delays in diagnosis and treatment are common [3]. Identification of bone marrow oedema on MRI is of importance for showing inflammation of the sacroiliac joints and supports diagnosis of axial SpA [4–7]. This directly influences the decision to treat patients with disease-modifying or biologic drugs [5].

Unfortunately, the definition of active inflammation on MRI is based on subjective criteria and is heavily dependent on the expertise and opinion of the scan reader [8–11]. ‘Conventional’ MR images used to detect inflammation—typically short inversion time inversion recovery (STIR) and T1-weighted spin echo images—produce complex image contrast that depends on multiple tissue properties, including T1, T2, proton density, perfusion and diffusion [11–13], which may confound the identification and quantification of oedema. These factors can lead to a lack of consistency between observers and scanners/hospitals [7, 14]. Therefore, there is a need for a method which can simply and objectively assess skeletal inflammation on MRI scans to support diagnostic and therapeutic decisions.

Previous studies have investigated the use of diffusion-weighted imaging (DWI) and chemical shift-encoded MRI (CSE-MRI) as objective methods for assessing bone marrow oedema, with promising initial results [12, 13, 15]. Using DWI, it has been shown that apparent diffusion coefficient (ADC) measurements are increased in areas of marrow oedema, probably due to an expansion of the extracellular space [13, 15–17]. Using CSE-MRI, it has been shown that proton density fat fraction (PDFF) measurements are reduced in areas of oedema compared with normal marrow, due to increased water content [12]. CSE-MRI can also be used to assess the severity of fat metaplasia, defined as a focal increase in content in areas of previous inflammation (with diagnostic and prognostic significance), in a quantitative fashion [12]. Previous studies measuring ADC in subchondral bone have typically relied on manual placement of regions-of-interest (ROIs) within the subchondral bone [15, 16, 18] which introduces substantial methodological subjectivity. Furthermore, these studies have relied on *mean* ADC measurements, which may perform poorly in patients with mixed active and chronic inflammation due to neutralisation of opposing effects [19]. There is currently no validated tool for quantifying proton density fat fraction (PDFF) in the sacroiliac joints.

We describe a new analysis tool which enables a more complete and consistent assessment of subchondral bone and derives a series of histographic parameters from both ADC and PDFF maps, aiming to isolate and separately quantify the active and chronic components of the inflammatory process. We aimed to demonstrate proof-of-principle for this tool in a prospective study of young people with SpA.

## Methods

This study received ethical approval from the Queen Square Research Ethics Committee, London, UK (Research Ethics Committee reference 15/LO/1475). All participants gave written informed consent prior to study entry.

### Study design and participants

A prospective cross-sectional study was performed at a single specialist tertiary referral centre for adolescents and young adults with inflammatory arthritis. Fifty-three consecutive patients meeting the eligibility criteria (mean age, 18 years; age range, 12–23 years) were prospectively recruited between July 2016 and December 2018 (31 males, mean age 18 years, and 22 females, mean age 17 years). Patients were included if they were referred for an MRI scan of the sacroiliac joints for suspicion of sacroiliitis or for monitoring of known sacroiliitis and were excluded if they had a contraindication to MRI scanning. All patients with known, pre-existing sacroiliitis had a clinical diagnosis of either non-radiographic axial SpA or enthesitis-related arthritis [18–21]. The sample size was fixed and based on logistical constraints. Patients were classified according to the presence or absence of bone marrow oedema and fat metaplasia using established criteria, based on conventional MRI scans, as described below.

### Image acquisition

All subjects underwent both quantitative and conventional MRI scans on the same visit. Quantitative CSE-MR images were acquired on a 3-T Philips Ingenia scanner (Ingenia, Philips) using an investigational version of the Philips mDixon Quant acquisition and post-processing pipeline, as described previously [12]. The images were acquired using a multi-echo gradient echo acquisition with bipolar readout (TE_1_ 1.17 ms, ΔTE 1.6 ms, TR 25 ms, flip angle 3°, matrix size 320 × 320, pixel spacing 1.76 × 1.76 mm, bandwidth 394 Hz/Px) and PDFF maps were generated using complex fitting incorporating T2* decay and a 10-peak model of human adipose tissue [12]. Images were acquired coronal to the long axis of the sacroiliac joint [12]. DW images were acquired on a 1.5-T Siemens Avanto scanner (Avanto, Siemens) using *b* values of 0, 50, 100, 300 and 600 s/mm^2^ with spectrally attenuated inversion recovery (SPAIR) fat suppression and echo planar imaging readout (TE = 89 ms, TR = 3600 ms, 4 averages, 8 mm slices, matrix size 144 × 192, FOV 237 × 316 mm, bandwidth 1447 Hz/Px), with images acquired axial to the sacroiliac joint [13, 15]. Conventional MRI consisted of T2-weighted STIR images, T1-weighted turbo spin echo images and fat-suppressed post-contrast T1-weighted turbo spin images acquired coronal to the sacroiliac joint (see Supplementary Information for sequence parameters) [11, 12].

### Image analysis

**Fig. 1:**
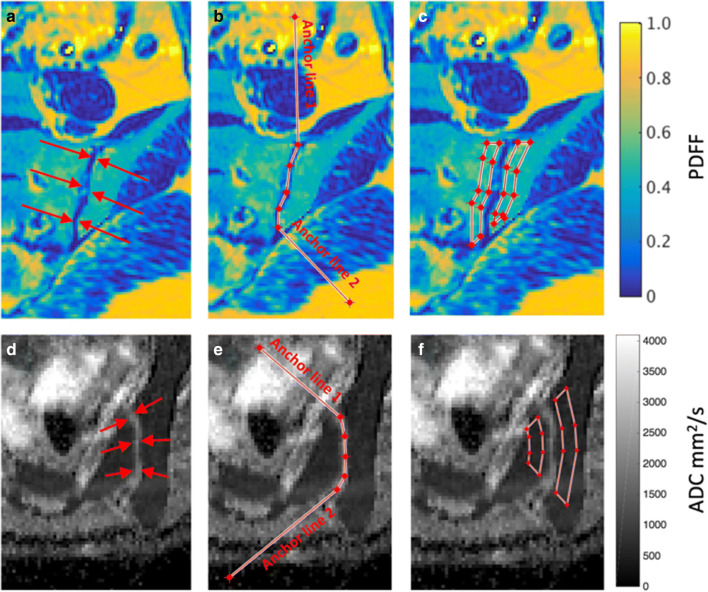
Definition of polygonal ROIs on subchondral bone. The observer is asked to define the line of the sacroiliac joint and ‘anchor lines’ are added to define the angles made by the joint with the anterior and posterior cortex of the bone, thus enabling the automatically propagated ROIs to better fit the subchondral bone

**Fig. 2:**
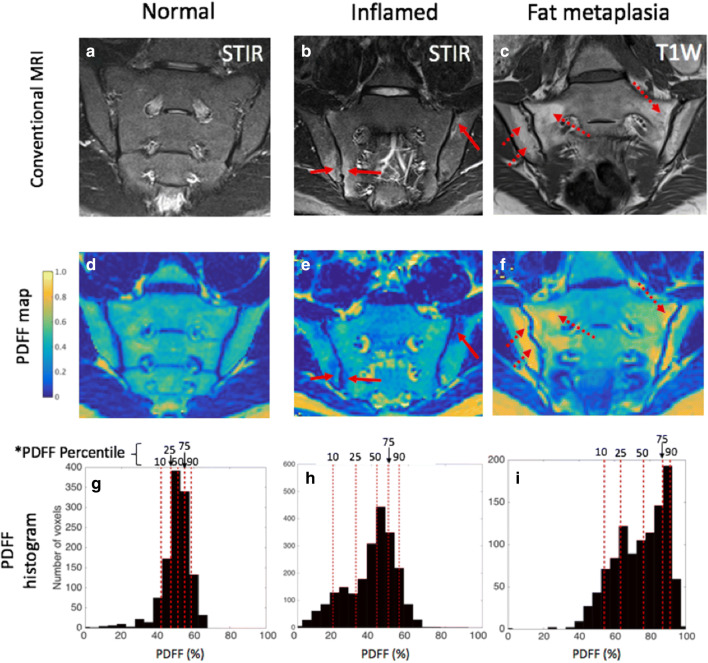
Examples of histograms generated using the BEACH tool. Conventional MR images (**a**–**c**), PDFF maps (**d**–**f**) and PDFF histograms (**g**–**i**) are shown. In the normal patient’s histogram (**g**), PDFF values are clustered around 50%, corresponding to normal marrow. In the patient with inflammation, a number of low-PDFF pixels have emerged in the histogram (**h**). In the patient with fat metaplasia, there is an upward shift in PDFF values, with a large number of high-PDFF pixels (**i**)

**Fig. 3:**
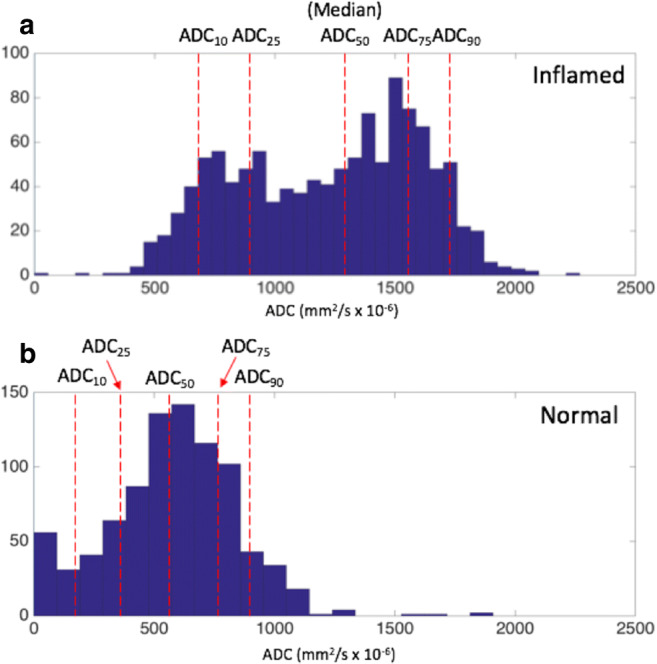
Examples of ADC histograms in patients with sacroiliitis (**a**) and control patients (**b**). The red lines indicate the 10th, 25th, 50th, 75th and 90th percentiles of the ADC distribution

Histographic parameters were obtained from the PDFF and ADC maps used an in-house software tool known as BEACH (Bone Edema and Adiposity Characterisation with Histograms) as shown in Figs. 1, 2 and 3, and as described in detail in the Supplementary Information. This method generates a series of histographic parameters for both ADC and PDFF.

The BEACH tool operates as follows. The observer is prompted to define the line of the sacroiliac joint using a single series of connected straight lines—an open polygon (Fig. 1). ‘Anchor lines’ are used to define the angle made by the joint with the cortical surface, at both the top and bottom of the joint, enabling the shape of the polygonal ROIs to be closely matched to subchondral bone. The software automatically generates a pair of polygonal ROIs in the subchondral bone either side of the joint (Fig. 1, Supplementary Figure S1). This is repeated for both sacroiliac joints covering the entire fibrocartilaginous part of the joint. For the ADC maps, all slices where the fibrocartilaginous joint was visible were included, whereas alternate slices were used for the PDFF maps due to the smaller slice thickness. For each patient, pixel values from the total volume of defined subchondral bone (i.e. from all ROIs) are analysed histographically. For both PDFF and ADC, we measured the 10th, 25th, 50th, 75th, and 90th centiles of the distribution (designated PDFF_10_, PDFF_25_… and ADC_10_, ADC_25_.. etc., as shown in Figs. 2 and 3). For each quantitative score, the mean of the two observers’ measurements was used for analysis.

The BEACH analysis was performed by two radiology residents (NS and AD, with 2 and 1 year of experience in MR imaging) who received a detailed training session (from TB). Both residents were blinded to all clinical information and to the qualitative radiological scores.

### Scoring of conventional MRI

Each subject’s set of conventional MR images was scored by two experienced musculoskeletal radiologists (KR and MHC) with 10 and over 25 years of MRI experience, both blinded to clinical diagnosis, to treatment and to the quantitative image data. Images were read on a research workstation. For each patient, observers assigned a qualitative score between 0 and 72 for the extent/severity of bone marrow oedema [22]. The patient was deemed to have active inflammation if the mean bone marrow oedema score from the two readers was ≥ 2, as per the Assessment of SpondyloArthritis Internal Society (ASAS) criteria [9, 23, 24]. Structural lesions consisting of fat metaplasia, erosions and joint ankylosis were assessed using a structural visual scoring system [25]. Patients with a score of ≥ 3 were deemed to be positive for the presence of fat metaplasia [25, 26].

### Clinical scores

Symptoms were assessed using a dedicated research questionnaire (see Supplementary Information). We report here the Bath Ankylosing Spondylitis Disability Index (BASDAI) and Bath Ankylosing Spondylitis Functional Index (BASFI), in addition to C-reactive protein (CRP) and erythrocyte sedimentation rate (ESR).

### Statistical analysis

Quantitative parameters derived from ADC and PDFF maps were compared between groups with and without inflammation/fat metaplasia using logistic regression (∝ = 0.05) and receiver-operating characteristic analyses. The optimal operating point for the ROC analysis was defined as the cut-point with the highest positive likelihood ratio (LR+) producing sensitivity and specificity greater than 70%. ROC-AUC values for percentile measurements were compared against the median using the method of DeLong et al [27], implemented using the *roccomp* function in Stata (∝ = 0.05). To evaluate whether combinations of parameters could improve prediction, multiple logistic regression was performed using combinations of ADC-based and PDFF-based parameters. Likelihood ratio testing was used to test whether combinations of explanatory variables provided an improved fit. Linear regression was used to evaluate the relationship between the qualitative scores and the best-performing qMRI parameters from the ROC analysis. Spearman correlation was used to evaluate the relationship between clinical scores and radiological scores. Inter- and intra-observer variability was assessed using the Bland-Altman 95% limits of agreement and the intra-class correlation coefficient.

## Results

### Detection of inflammation

Fifteen of 53 patients (24.5%) had sufficient bone marrow oedema to meet the ASAS criteria for active inflammation. The inflamed group included 12 males and 3 females. The gender difference between the inflamed and uninflamed groups was not significant (*p* = 0.065). There was no significant age difference between the inflamed and uninflamed groups (*p* = 0.43).

**Fig. 4:**
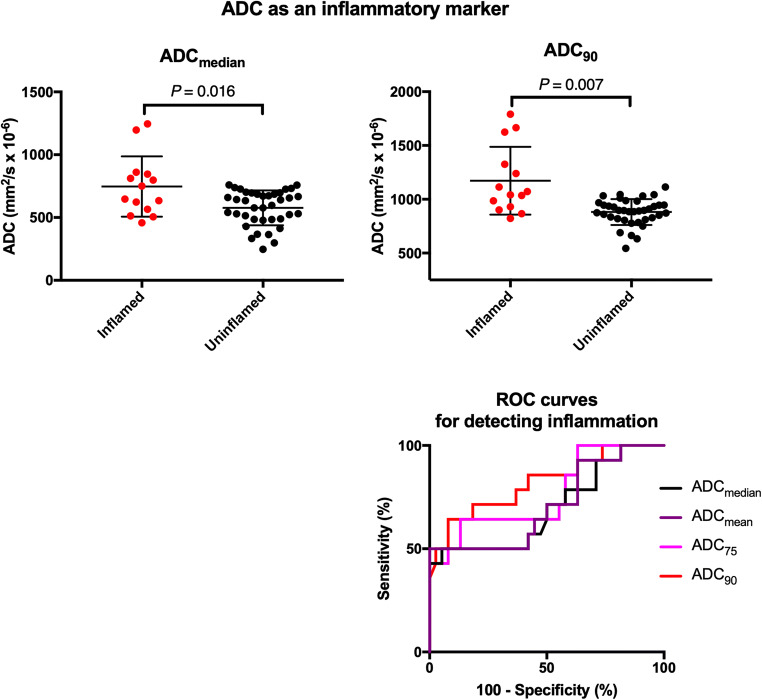
ADC as an inflammatory marker. Representative BEACH parameters (ADC_median_ and ADC_90_) are compared between inflamed and uninflamed groups. The displayed *p* values were obtained by logistic regression. ROC curves for all relevant parameters are shown in the bottom right

**Fig. 5:**
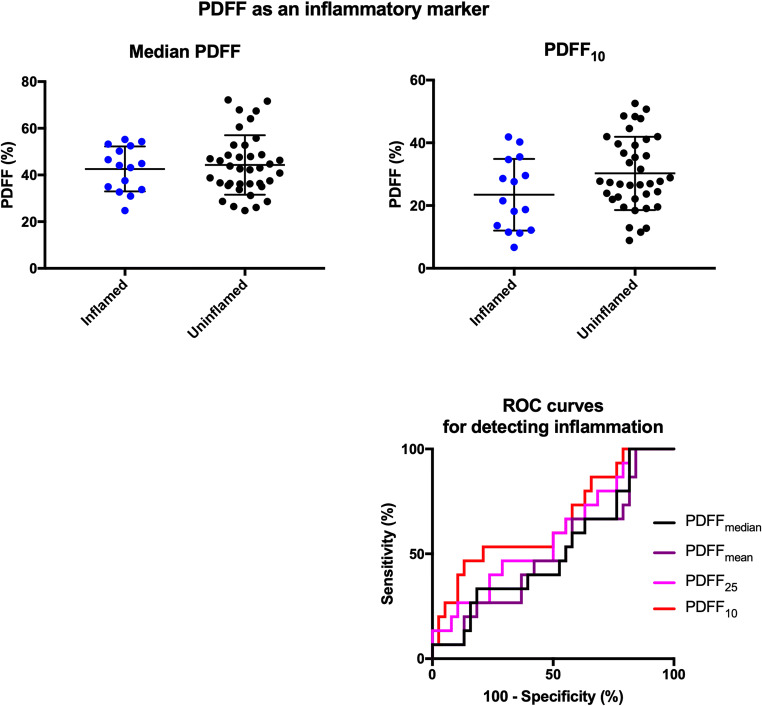
PDFF as an inflammatory marker. Representative BEACH parameters (PDFF_median_ and PDFF_10_) are compared between inflamed and uninflamed groups. The displayed *p* values were obtained by logistic regression. ROC curves for all relevant parameters are shown in the bottom right

Comparisons of quantitative parameters between inflamed and uninflamed SIJs are shown in Figs. 4 and 5, and the results of the corresponding logistic regression and ROC analyses are shown in Table 1.

**Table 1:** Comparison of inflammatory parameters between inflamed and non-inflamed patients. ADC_75_, ADC_90_, etc. refer to the 75th and 90th percentiles of ADC measurements in the defined ROI. Estimates from each group are displayed as mean (95% CI). Odds ratio (OR) and *p* values (*) were derived from logistic regression. The highest ROC AUC value for the evaluation of inflammation is shown in italics. Sensitivity and specificity values for the optimal cutoff values (far right) are provided in the main “Results” section. The right-hand *p* values (**) relate to the comparison of ROC AUC with the median value

	Inflammation						
	Present (*n* = 15)	Absent (*n* = 38)	OR (95% CI)	*p=	ROC AUC (95% CI)	Optimal cutoff	**p=
ADC_mean_	758 (629–887)	578 (535–621)	1.006 (1.001–1.011)	0.011	0.709 (0.530–0.888)	–	0.249
ADC_median_	747 (621–872)	577 (534–620)	1.006 (1.001–1.011)	0.016	0.695 (0.512–0.877)	–	–
ADC_75_	964 (813–1115)	733 (695–770)	1.008 (1.002–1.014)	0.011	0.763 (0.602–0.924)	–	0.073
ADC_90_	1172 (1007–1337)	882 (842–921)	1.010 (1.002–1.017)	0.007	*0.819* (0.676–0.962)	986	0.072
PDFF_mean_	42.3 (37.2–47.4) %	43.9 (40.0–47.8) %	0.988 (0.934–1.041)	0.650	0.505 (0.329–0.682)	–	0.640
PDFF_median_	42.6 (37.8–47.6) %	44.3 (40.2–48.4) %	0.988 (0.937–1.040)	0.639	0.514 (0.337–0.691)	–	–
PDFF_25_	32.8 (27.5–38.1) %	37.1 (33.4–40.8) %	0.964 (0.910–1.021)	0.215	0.589 (0.414–0.763)	–	0.010
PDFF_10_	23.5 (17.8–29.2) %	30.3 (26.6–34.0) %	0.948 (0.895–1.004)	0.067	0.657 (0.485–0.829)	–	0.001

All ADC-based parameters were associated with significantly increased odds of inflammation. Parameters which sampled the upper end of the ADC distribution (i.e. ADC_75_ and ADC_90_) performed best for distinguishing inflamed from uninflamed SIJs; ADC_90_ produced an AUC value of 0.819 (0.676–0.962; *p* = 0.072 when compared with ADC_median_). The optimal cutoff for ADC_90_ was 986 mm^2^/s (sensitivity 71.4%, specificity 81.6%). Cutoffs for ADC_mean_, ADC_median_ and ADC_75_ did not meet pre-specified performance thresholds.

PDFF-based parameters performed poorly as measures of inflammation with no significant difference between inflamed and uninflamed SIJs. Nonetheless, performance increased for parameters sampling the lower end of the distribution (AUC = 0.657 for PDFF_10_, 0.514 for PDFF_median_).

### Detection of fat metaplasia

Thirty of 53 patients (56.6%) met the criteria for fat metaplasia. Patients with fat metaplasia were significantly older than those without fat metaplasia (mean ages (95% CI) were 19.6 (18.5–20.7) and 17.6 (16.5–18.7) respectively (*p* = 0.046)). There was no significant difference in gender between patients with and without fat metaplasia (*p* = 0.56).

**Fig. 6:**
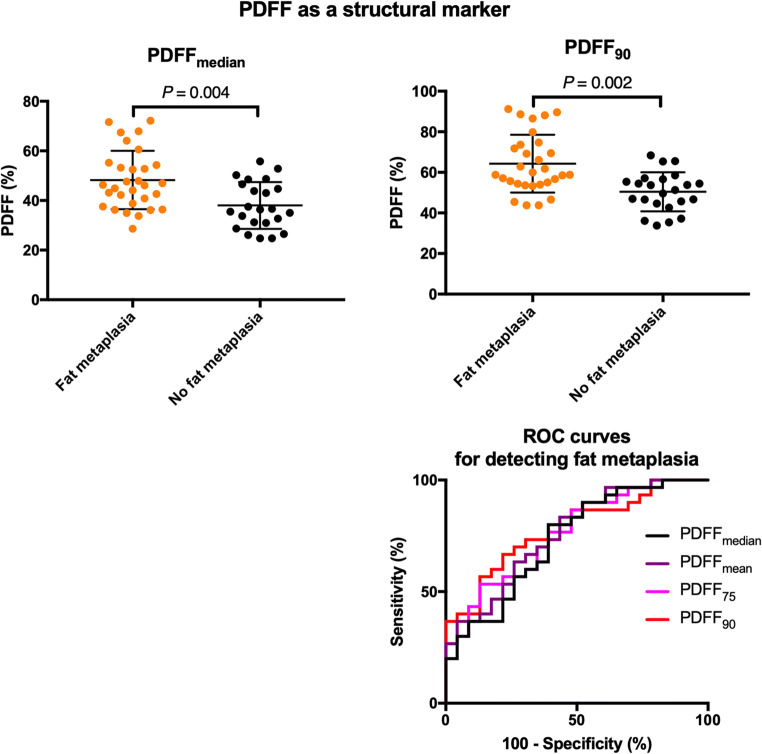
PDFF as a structural marker. Representative BEACH parameters (PDFF_median_ and PDFF_90_) are compared between patients with and without fat metaplasia. The displayed *p* values were obtained by logistic regression. ROC curves for all relevant parameters are shown in the bottom right

Comparisons of quantitative parameters between patients with and without fat metaplasia are shown in Fig. 6, and the results of the corresponding logistic regression and ROC analyses are shown in Table 2.

**Table 2:** Comparison of structural parameters between patients with and without fat metaplasia. PDFF_75_, PDFF_90_, etc. refer to the 75th and 90th percentiles of ADC measurements in the defined ROI. Estimates are displayed as mean (95% CI). Odds ratio (OR) and *p* values (*) were derived from logistic regression. The highest ROC AUC value for the evaluation of fat metaplasia is shown in italics. Sensitivity and specificity values for the optimal cutoff values (far right) are provided in the main “Results” section. The right-hand *p* values (**) relate to the comparison of ROC AUC with the median value

	Fat metaplasia							
	Present (*n* = 30)	Absent (*n* = 23)	OR (95% CI)	*p=	ROC AUC (95% CI)	Optimal cutoff	**p=	
PDFF_mean_	48.2 (43.9–52.5) %	37.4 (33.7–41.1) %	1.110 (1.035–1.119)	0.003	0.759 (0.629–0.889)	–	0.061	
PDFF_median_	48.2 (44.1–52.3) %	38.0 (34.0–41.9) %	1.100 (1.030–1.174)	0.004	0.735 (0.597–0.872)	–	–	
PDFF_75_	57.1 (52.0–62.2) %	44.5 (48.2–40.8) %	1.105 (1.034–1.182)	0.003	0.759 (0.630–0.888)	–	0.254	
PDFF_90_	64.3 (59.2–69.4) %	50.4 (46.5–54.3) %	1.111 (1.037–1.189)	0.002	*0.780* (0.656–0.903)	55.7%	0.263	
ADC_mean_	606 (541–671)	652 (571–732)	0.999 (0.996–1.002)	0.380	0.567 (0.407–0.726)	–	0.669	
ADC_median_	602 (535–669)	648 (572–724)	0.999 (0.996–1.002)	0.380	0.572 (0.413–0.731)	–	–	

PDFF-based parameters were associated with increased odds of fat metaplasia, and the separation between patients with and without fat metaplasia was improved for parameters which specifically sampled the upper end of the PDFF distribution (i.e. PDFF_75_ and PDFF_90_). The best performing parameter, PDFF_90_, had an AUC of 0.780 (0.656–0.903; *p* = 0.263 when compared with PDFF_median_). The optimal operating point for PDFF_90_ was 55.7%, producing a sensitivity of 70% and a specificity of 73.9%.

There were no significant differences in ADC_mean_ or ADC_median_ between patients with and without fat metaplasia.

### Prediction of inflammation and fat using combinations of parameters

Multiple logistic regression using both ADC_90_ and FF_90_ or ADC_90_ and FF_median_ as predictor variables did not significantly improve the model fit compared with simple logistic regression using ADC_90_ as a single predictor (*p* = 0.41 and 0.81, respectively). Similarly, the combination of FF_90_ and ADC_90_ or FF_90_ and ADC_median_ did not improve the model fit compared with using FF_90_ alone (*p* = 0.86 and 0.73, respectively).

### Relationship between BEACH parameters and qualitative MRI scores

The relationship between visual scores of inflammation/fat metaplasia and qMRI parameters is shown in Supplementary Figure S2. There were significant positive relationships between ADC_90_ and the visual inflammation score (slope = 15.33, *p* < 0.0001) and between PDFF_90_ and the fat metaplasia score (slope = 1.05, *p* < 0.0001).

### Relationship between MRI and symptoms

Scatterplots showing the relationship between BASDAI scores and visual and quantitative scores of inflammation and fat metaplasia are shown in Supplementary Figure S3.

There was no significant correlation between visual scores of inflammation and any clinical score (*p* = 0.45, 0.48, 0.14 and 0.49 for BASDAI, BASFI, CRP and ESR) or between ADC_90_ parameters and clinical scores (for ADC_90_ *p* = 0.48, 0.37, 0.19 and 0.63).

There was a significant negative relationship between fat metaplasia visual scores and clinical symptoms (*p* = 0.004 and 0.006 for BASDAI and BASFI), and a similar relationship was observed for the corresponding qMRI parameter PDFF_90_ (*p* = 0.03 and 0.01 for BASDAI and BASFI). There was no significant relationship between either visual or quantitative fat metaplasia scores and CRP or ESR (all *p* > 0.05).

### Inter- and intra-observer agreement

Inter- and intra-observer agreement statistics for qMRI parameters and visual scores are shown in Table 3. Inter-observer and intra-observer agreement were excellent for all assessed qMRI parameters. Inter-observer agreement was excellent for visual inflammation scores, although the 95% limits of agreement (0.6 ± 6.4) were relatively wide compared with the ASAS definition of active inflammation (score of ≥ 2 diagnostic for active inflammation). Inter-observer agreement was poorer for fat metaplasia scores with an ICC value of 0.544.

**Table 3:** Inter-observer and intra-observer variability statistics for selected (most relevant) parameters. The intra-class correlation coefficient and Bland-Altman limits of agreement are shown

	Inter-observer variability		Intra-observer variability (observer 1)		Intra-observer variability (observer 2)	
	ICC	95% LoA	ICC	95% LoA	ICC	95% LoA
ADC_mean_	0.962	8.4 ± 103 mm^2^/s	0.949	− 27.6 ± 128 mm^2^/s	0.949	− 0.19 ± 112 mm^2^/s
ADC_median_	0.943	8.4 ± 111 mm^2^/s	0.954	− 20.6 ± 124 mm^2^/s	0.898	11 ± 156 mm^2^/s
ADC_90_	0.918	− 10.1 ± 188 mm^2^/s	0.918	− 38.1 ± 210 mm^2^/s	0.961	3.9 ± 123 mm^2^/s
PDFF_mean_	0.982	− 1.03 ± 4.42%	0.994	0.15 ± 2.40%	0.986	− 1.08 ± 3.69%
PDFF_median_	0.974	− 0.97 ± 5.08%	0.989	0.34 ± 3.20%	0.968	− 0.87 ± 5.45%
PDFF_90_	0.986	− 0.31 ± 4.70%	0.990	− 0.41 ± 3.60%	0.968	0.05 ± 6.52%
Visual inflammation score	0.944	0.6 ± 6.4	–	–	–	–
Visual fat metaplasia score	0.534	− 6.8 ± 18.3	–	–	–	–

## Discussion

We describe a quantitative, partially automated method for measurement of bone marrow oedema and fat metaplasia based on histographic analysis of quantitative MR images. We show that histogram-based qMRI parameters enable separation of patients according to the presence of oedema and fat metaplasia, both of which are of importance for the diagnosis and management of SpA. The proposed tool offers a simple and potentially repeatable means to quantify inflammation and fat and could be incorporated into picture archiving and communications system (PACS) systems relatively easily. Such a tool could be of value for monitoring inflammation over time and for guiding clinical decisions around initiation and changes of biologic and other therapies. Importantly, ADC-based and PDFF-based parameters provide discrete information regarding oedema and fat metaplasia and could therefore inform on the relative burden of active inflammation versus structural damage.

We found that ADC measurements produced superior performance to PDFF measurements for separating patients with and without inflammation. This suggests that increases in diffusivity are an important part of the inflammatory process in the bone marrow, rather than changes in water content per se. However, previous studies have shown substantial differences in PDFF between normal and inflamed marrow [12], and it may be that the discrepant observations in this study are due to the variability in the composition of normal bone marrow [28]. This could be investigated further by comparing the composition of the inflamed subchondral bone with normal bone marrow.

Our results showed that PDFF_90_ enabled separation of patients with and without fat metaplasia. Fat metaplasia can contribute to diagnosis [6, 7] and is also a prognostic factor, since patients with fat metaplasia are more likely to fuse their sacroiliac joints [29–31].

Interestingly, the 90th percentiles of ADC and PDFF yielded more accurate separation of inflamed and non-inflamed joints and joints with and without fat metaplasia compared with simple averages, although this difference did not reach statistical significance. This suggests that percentiles measuring the extremes of the distribution might be better ‘targeted’ to areas of oedema (for ADC) or fat metaplasia (for PDFF) than mean or median measurements, which may be ‘contaminated’ by non-inflamed or non-fatty sites, respectively, to a greater extent.

Importantly, the inter- and intra-observer variability for both ADC- and PDFF-based parameters was good or excellent. Inter-observer variability was excellent for visual scoring of bone marrow oedema, but substantially poorer for scoring of fat metaplasia. Given the known inconsistencies in radiologists’ interpretation in spondyloarthritis in clinical practice [14], a more consistent measurement could be a major advantage. However, formal studies are needed to assess repeatability and reproducibility across sites and MRI vendors.

We did not find a strong relationship between inflammation on MRI and symptoms in this study, likely reflecting the complex and multidimensional nature of pain in SpA [32]. There was a negative relationship between the severity of fat metaplasia and symptom scores. This suggests that fat metaplasia, a post-inflammatory phenomenon [30, 33], is more common in patients already on treatment with well-controlled symptoms.

A strength of our study is that the control subjects (i.e. those without inflammation) were patients where MRI was clinically indicated and thus likely to have either biomechanical back pain or quiescent inflammatory arthritis. Consequently, the reported statistics for separating patients with and without inflammation are likely to be realistic in a real-world clinical setting (this point is emphasised in the QUADAS-2 quality criteria [34]). By contrast, the use of healthy controls can artificially inflate sensitivity and specificity statistics and give a misleading impression of diagnostic performance. An additional strength is that the histographic parameters used are relatively simple and likely to offer superior performance to more complex metrics based on maximum likelihood estimation. Nonetheless, future work could explore the use of more complex analysis methods, such as Gaussian mixture modeling, to identify discrete subpopulations of pixels within the ROI.

A limitation of this study is that the diagnostic performance reported is not likely to be sufficient for the current use in clinical practice. This may be partially due to the variations in the composition of normal marrow in young patients, where the marrow may be partially ossified and contains varying proportions of water and fat. This factor may bias ADC and PDFF measurements and could have weakened the separation of inflamed and non-inflamed patients. In the future, the BEACH tool could be extended to isolate ossified bone, potentially improving performance. Similarly, the proportion of red and yellow marrow in ossified bone may vary between individuals. The use of variable thresholds depending on the composition of the normal ‘background’ marrow might help to improve the technique for detecting inflammation. ADC measurements can also suffer from poor reproducibility across sites, partly due to the difficulty of achieving high-quality fat suppression [19]. A final limitation is that the proposed tool is only partially automated; further methodological development is required to achieve full automation.

In conclusion, we describe a method for quantifying bone marrow oedema and fat metaplasia in patients with SpA, based on histographic analysis. ADC-based parameters can objectively differentiate patients with bone marrow oedema from those without, whilst PDFF-based parameters can differentiate patients with fat metaplasia from those without. Histographic analysis might improve performance compared with simple averages such as the mean and median and offers excellent agreement within and between observers.

## Electronic supplementary material

ESM 1 (DOCX 1819 kb)

#### Generation of polygonal ROIs

The observer is prompted to define the line of the sacroiliac joint using a single series of connected straight lines - an open polygon – as shown in Fig. S1. This open polygon includes ‘anchor lines’ (shown in blue), which define the angle made by the joint with the cortical surface, at both the top and bottom of the joint (Fig. S1), and the line of the joint itself. The first anchor line is placed perpendicular to the surface of the bone at the upper end of the sacroiliac joint, in order to define the angle α between the bone surface (labeled *s*) and the joint itself. Next, the observer places a series of joint lines (also shown in blue, labeled j_1_… j_n_), which trace the course of the synovial part of the sacroiliac joint. Finally, the observer places a second anchor line defining the angle β formed between the joint and the cortical surface (in the case of axial diffusion-weighted images, the ligamentous part of the joint is not including in the scoring so the angle β is formed between the synovial and ligamentous parts of the joint, rather than at the cortical surface). A series of lines (shown in red on Fig. S1a) with width w_1_ are then automatically generated and placed such that each line is perpendicular to the preceding joint line. In this study, we used w_1_ = 14 pixels (equal to 21.8 mm for PDFF maps and 23.4 mm for ADC maps), chosen empirically. The coordinates of these lines are used to form a polygonal ROI including both the subchondral bone and joint space (on Fig. S1b). This procedure is repeated to generated a further set of shorter perpendicular lines_,_ with width w_2_, which define the width of the joint space itself (not shown). Here, we used w_2_ = 6 pixels (equal to 9.36 mm for PDFF maps and 10.02 mm for ADC maps). Finally, the coordinates of these two sets of lines are used to generate two separate polygonal areas A_1_ and A_2_ adjacent to the joint, which comprise the total region-of-interest A for a single SIJ (Fig. S1c).

#### Clinical Scores

For each patient, symptoms were assessed using a dedicated research questionnaire designed by an adolescent and young adult rheumatologist (XX), which included the Bath Ankylosing Spondylitis Disability Index (BASDAI), Bath Ankylosing Spondylitis Functional Index (BASFI), Health Activity Questionnaire (HAQ), Work Productivity Impairment Questionnaire plus Classroom Impairment Questions (WPAI+CIQ), Patient Global Assessment, Jenkin’s Sleep scale and Margolis Pain Diagram. Additionally, all patients were clinically examined by a specialist adolescent rheumatologist at the time of scan referral to determine the presence of swollen, restricted or active joints, and of enthesitis.

#### Conventional MRI

Conventional MRI consisted of T2-weighted STIR images acquired coronal to the sacroiliac joint using a turbo inversion recovery magnitude (TIRM) sequence (TE 77 ms, TR 3880 ms, TI 150 ms, 2 averages, matrix size 256 × 192, FOV 200x200mm, 4 mm slices, bandwidth 132 Hz/Px, echo train length 11) and T1-weighted turbo spin echo images (TE 9.2 ms, TR 687 ms, 2 averages, matrix size 256 × 243, FOV 200x200mm, 3 mm slices, bandwidth 171 Hz/Px, echo train length 5), with fat-suppressed T1-weighted turbo spin echo images (TE 9.2 ms, TR 792 ms, 2 averages, matrix size 256 × 230, FOV 200x200mm, 3 mm slices, bandwidth 171 Hz/Px, echo train length 5) acquired after gadolinium injection, performed on the same 1.5 T Siemens Avanto scanner, again coronal to the sacroiliac joint [11, 12].

## Abbreviations

ADC: Apparent diffusion coefficient
AUC: Area under the curve
BASDAI: Bath Ankylosing Spondylitis Disability Index
BASFI: Bath Ankylosing Spondylitis Functional Index
BEACH: Bone edema and adiposity characterisation
BRC: Biomedical Research Centre
CRP: C-reactive protein
CSE-MRI: Chemical shift-encoded MRI
DWI: Diffusion-weighted imaging
ESR: Erythrocyte sedimentation rate
MRI: Magnetic resonance imaging
NIHR: National Institute for Health Research
OR: Odds ratio
PACS: Picture archiving and communications system
PDFF: Proton density fat fraction
qMRI: Quantitative MRI
ROC: Receiver operating characteristic
ROI: Region-of-interest
SIJ: Sacroiliac joint
SpA: Spondyloarthritis
STIR: Short inversion time inversion recovery
TNFi: Tumour necrosis factor inhibitor
