# Supplementary material for: Histographic analysis of oedema and fat in inflamed bone marrow based on quantitative MRI
Source: Eur Radiol. 2020 Apr 14;30(9):5099–109. doi: 10.1007/s00330-020-06785-x (PMC7431434; doi:10.1007/s00330-020-06785-x)

**Supplementary Materials**

Figure S1 – Geometry of ROIs propagated unto subchondral bone.


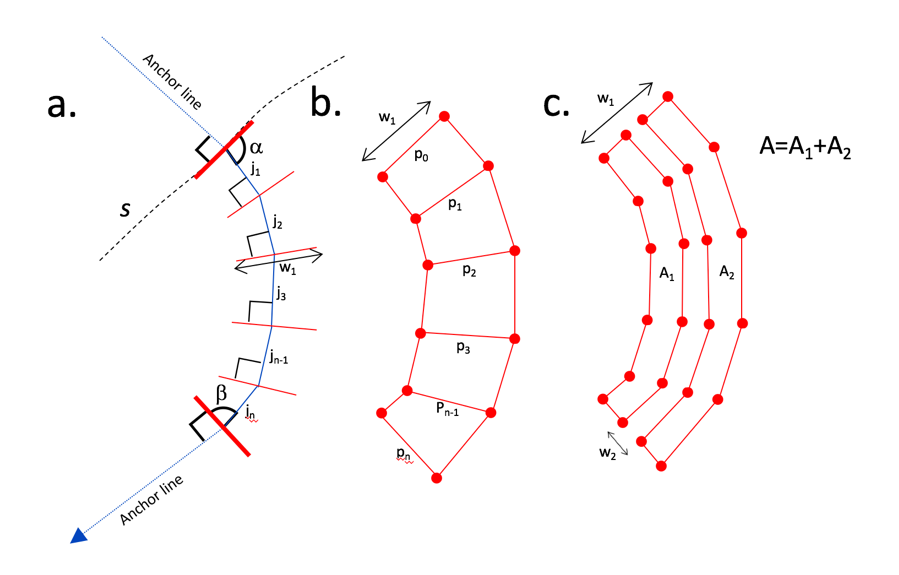


Figure S2 – Linear regression of qMRI parameters against visual inflammation scores.


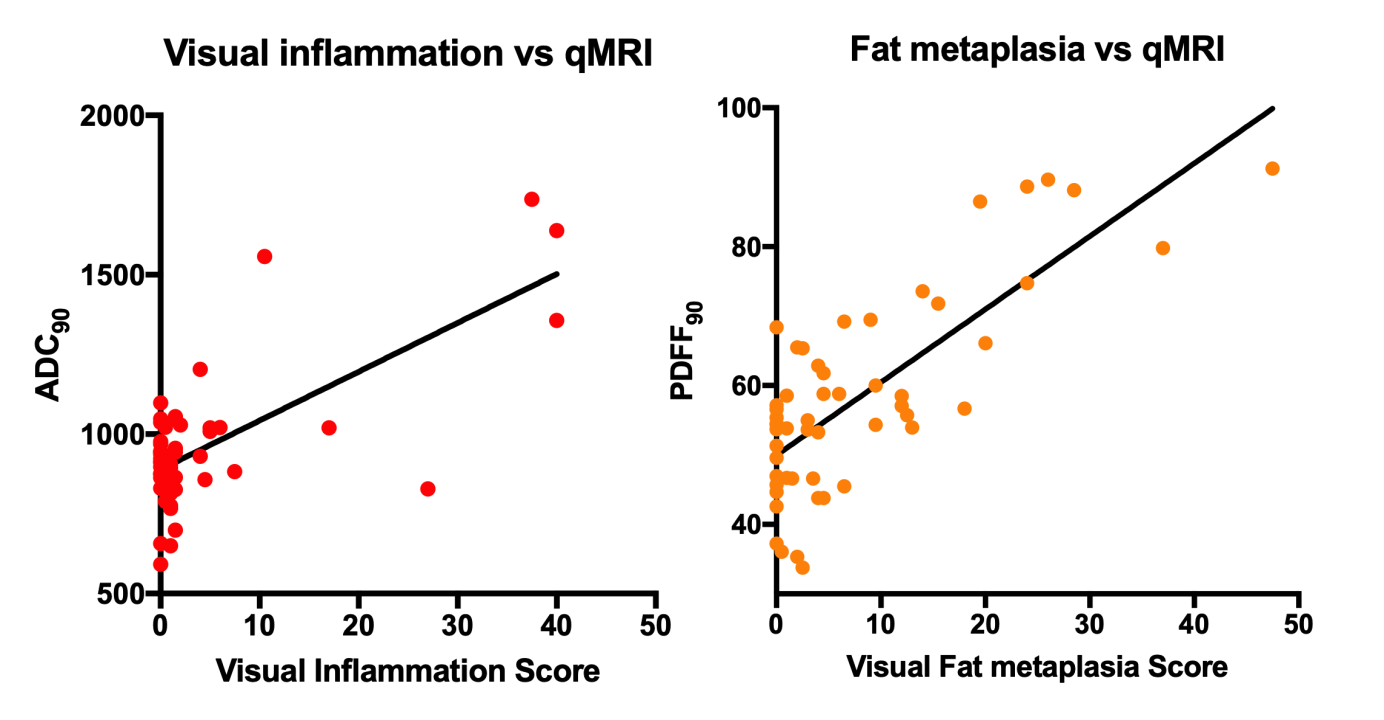


Figure S3 - Relationship between BASDAI symptom scores and imaging measures of inflammation and structural damage (fat metaplasia).


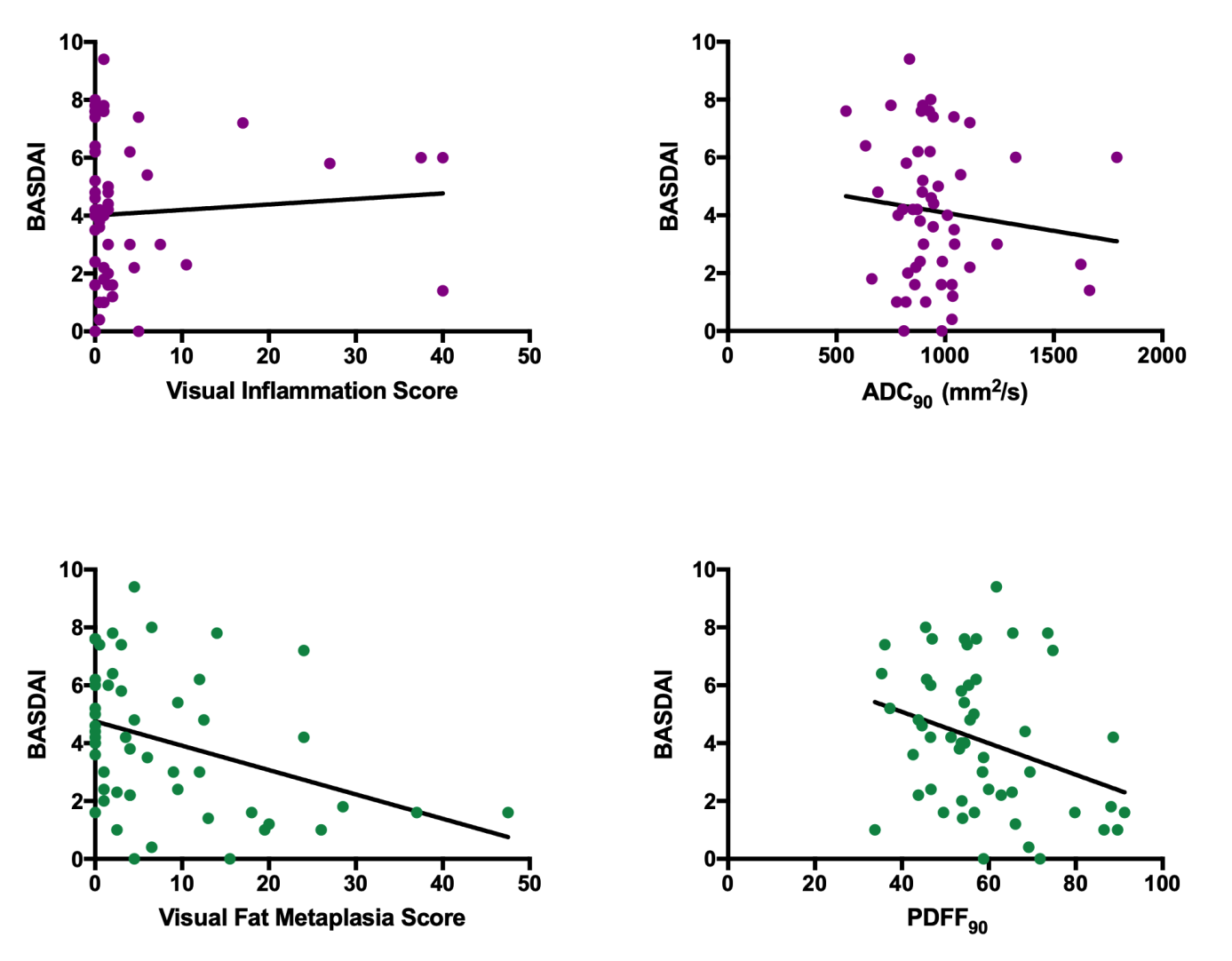

Supplement: Supplementary file 1 — (DOCX 1819 kb) [file 330_2020_6785_MOESM1_ESM.docx]
